# Pawpaw (*Asimina triloba*) Seed Extract Suppresses High-Fat Diet-Induced Obesity in Mice

**DOI:** 10.3390/ijms26167719

**Published:** 2025-08-09

**Authors:** Shiori Takano, Sakura Kaneko, Ryo Midorikawa, Honoka Nara, Yurie Sato, Minori Uchiyama, Haruka Iobe, Yuki Saito-Matsuzawa, Hideyuki Sone, Shin Kamiyama

**Affiliations:** 1 Department of Health and Nutrition, Faculty of Human Life Studies, University of Niigata Prefecture, 471 Ebigase, Higashi-ku, Niigata 950-8680, Japan; 2 Graduate School of Agricultural Science, Tohoku University, 468-1 Aramaki Aza Aoba, Aoba-ku, Sendai 980-8572, Japan; 3 Division of Health and Nutrition, Graduate School of Health and Nutrition, University of Niigata Prefecture, 471 Ebigase, Higashi-ku, Niigata 950-8680, Japan

**Keywords:** *Asimina triloba*, fat synthesis, high-fat diet, pawpaw seed extract, obesity

## Abstract

*Asimina triloba* (pawpaw), a member of the Annonaceae family, contains various bioactive phytochemicals, including alkaloids, polyphenols, and acetogenins. In this study, the effects of pawpaw seed extract (PSE) on obesity and plasma lipid concentrations were investigated in mice with high-fat diet (HFD)-induced obesity. Male C57BL/6J mice were fed a normal diet (ND) or an HFD for two weeks. The mice in the latter group were then divided into three groups: HFD, L-PSE, and H-PSE. Following a two-week adaptive period, the L-PSE and H-PSE groups were fed an experimental diet containing 250 mg and 500 mg PSE/kg of HFD, respectively, for two weeks. Mice in the HFD group exhibited significantly higher body weights than that of mice in the ND group. A significant decrease in body weight was observed in the H-PSE group compared with that in the HFD group. The perirenal, testicular, and total visceral fat masses of the mice in the H-PSE group were consistently lower than those of the mice in the HFD group. Administration of high-dose PSE decreased the expression of *Fasn* (encoding fatty acid synthase) and *Dgat2* (encoding diglyceride acyltransferase 2) in testicular fat tissues. However, PSE administration did not decrease blood glucose and plasma cholesterol levels compared with that in the HFD group. These findings suggest that the administration of PSE suppresses HFD-induced obesity in mice, while its hypoglycemic or cholesterol-lowering actions are less pronounced.

## 1. Introduction

*Asimina triloba* (pawpaw), a member of the Annonaceae family (also known as the custard apple family) native to eastern North America, produces an edible sweet fruit with a tropical flavor. Similarly to other species in the Annonaceae family, pawpaw has attracted substantial pharmacological interest owing to its high bioactive component content. The root, bark, seed, and fruit pulp contain alkaloids, including asiminine, analobine, and acimilobine, which have potential therapeutic effects [1]. Pawpaw also contains considerable amounts of various polyphenols [2]. Phenolic compounds in pawpaw extracts possess antioxidant and anti-inflammatory activities [2,3,4,5]. The antioxidant activities of water and alcoholic extracts of pawpaw trees and fruits are related to the content of hydrophilic phenolic compounds, including epigallocatechin, caffeine, catechin, chlorogenic acid, caffeic acid, epicatechin, epigallocatechin gallate, p-coumaric acid, gallocatechin gallate, ferulic acid, epicatechin gallate, rutin, catechin gallate, and naringin [2]. Additionally, a structurally diverse array of annonaceous acetogenins, a unique class of long-chain (C32 or C34) polyketide-derived fatty acid derivatives, was identified and investigated [6,7,8]. Acetogenins exhibit a wide range of bioactivities, including antimicrobial, antiviral, and pesticidal effects [6,9,10,11,12,13]. In particular, acetogenins exhibit potent cytotoxicity against various cancer cell lines, making them candidates for anticancer drug development [7,13,14].

Obesity is a major risk factor for many lifestyle-related diseases, including cardiovascular disease, diabetes mellitus, and hypertension. In the Annonaceae family, *Annona muricata*, more commonly referred to as graviola or soursop, has been used in traditional medicine due to its recognized anticancer, antihypertensive, antidiabetic, and hypolipidemic properties [15,16,17,18]. Graviola leaves are a source of polyphenolic antioxidants and often used for weight management [19,20,21]. Graviola contains a variety of bioactive compounds, including acetogenins, flavonoids, tannins, alkaloids, coumarins, and terpenoids [18,22,23,24]. Graviola-derived polyphenols exert beneficial effects on blood glucose regulation and lipid metabolism [18,22,25]. The 12-week administration of aqueous graviola leaf extract to obese C57BL/6 mice resulted in a dose-dependent reduction in body weight and plasma lipid levels [25]. Although pawpaw contains substantial polyphenol content analogous to that found in graviola [2,3,4,5,26], to the best of our knowledge, research on the functions of bioactive components on obesity and metabolic disorders was not conducted.

We previously reported that ethanolic pawpaw seed extract (PSE) inhibited adipocyte differentiation and triglyceride accumulation in 3T3-L1 cells, an experimental model of adipocyte differentiation and fat metabolism [27]. The PSE treatment significantly decreased the expression levels of regulators involved in adipocyte differentiation and adipogenesis, including proliferator-activated receptor γ (PPAR-γ), CCTTA/enhancer-binding protein (C/EBP)-α, and sterol regulatory element-binding protein (SREBP)-1c, under glucose restriction conditions [27]. Lee et al. also reported that 70% pawpaw fruit pulp ethanolic extract suppressed adipocyte differentiation and lipogenesis-related protein expression in 3T3-L1 cells [28]. However, whether PSE administration inhibits obesity and fat accumulation in vivo remains unclear. In the present study, we administered PSE to high-fat diet (HFD)-induced obese mice to evaluate its effects on obesity and lipid metabolism.

## 2. Results

### 2.1. Body Weight and Food Intake

On average, 200.4 mg of PSE was obtained from 1 g of grinded seeds. The concentration of total phenolic compounds in the PSE was determined to be 11.0 mg of gallic acid equivalent per g of PSE.

**Figure 1:**
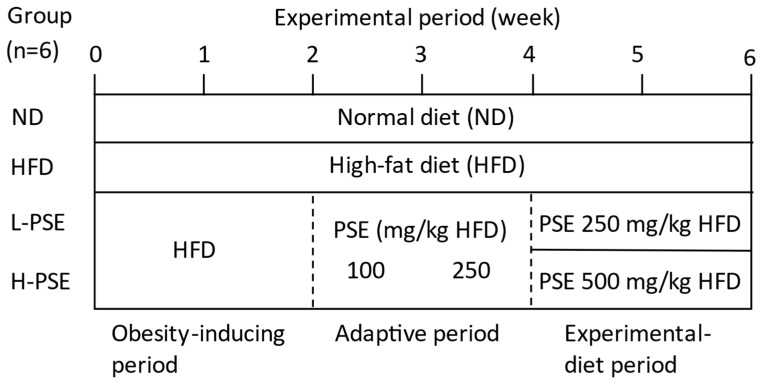
Schematic representation of the experimental design. Twenty-four mice were assigned to the normal diet (ND), high-fat diet (HFD), L-PSE (250 mg PSE/kg HFD), and H-PSE (500 mg/kg HFD) groups (*n* = 6 each). During the obesity-inducing period, mice in the HFD, L-PSE, and H-PSE groups were fed an HFD for two weeks. During the adaptive period, the PSE concentration was gradually increased (i.e., one week of 100 mg PSE/kg of the diet followed by one week of 250 mg/kg of the diet) to allow the mice to acclimatize to the taste of PSE. Subsequently, mice in the L-PSE and H-PSE groups were provided with their experimental diet as illustrated.

Two different concentrations of PSE were administered to HFD-induced obese mice: 250 mg/kg HFD (L-PSE group) and 500 mg/kg HFD (H-PSE group). Due to the possibility of the presence of astringent polyphenols and alkaloids in the seeds of the pawpaw, a two-week adaptive period was implemented prior to the two-week experimental diet-feeding period (Figure 1).

**Figure 2:**
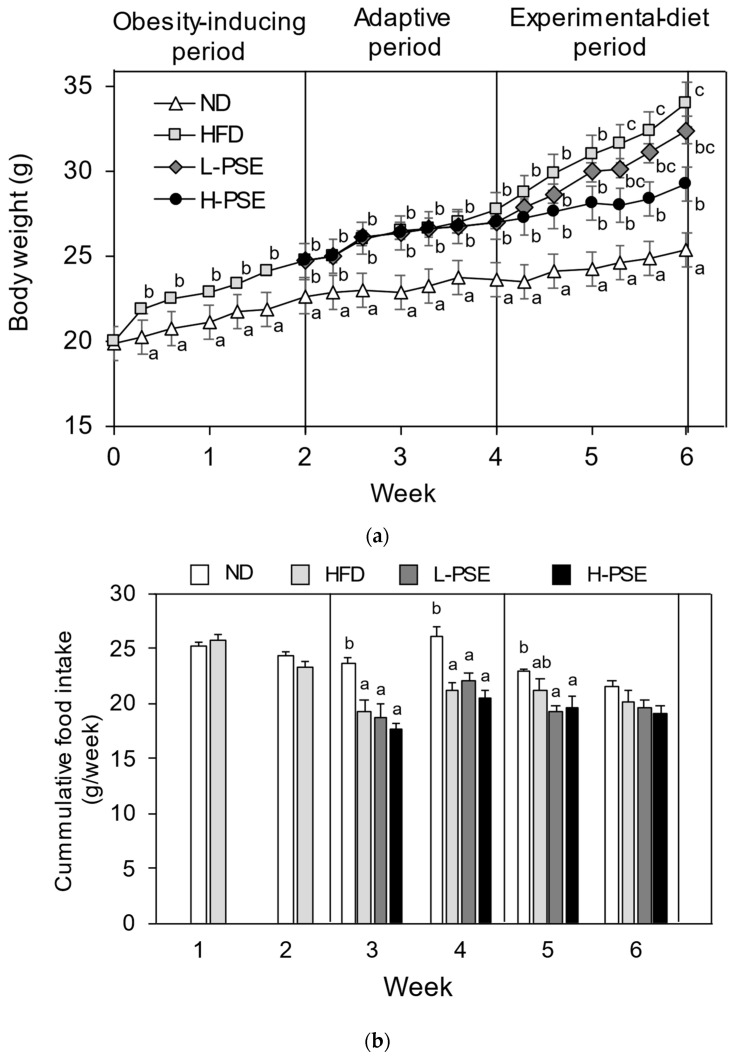
Body weight change (**a**) and cumulated food intake (**b**) of mice in each group during the experimental period. The initial (0) week of the experimental period corresponds to 5 weeks of age. Cumulative food intake was determined from the daily food intake. Values obtained from six mice are shown as mean ± SE. ^a,b,c^ Different letters indicate significant differences between values (*p* < 0.05, ANOVA followed by Tukey’s test). The absence of letters indicates that no significant differences were observed.

Figure 2a shows the change in body weight of the mice in each group during the obesity-inducing, adaptive, and subsequent experimental diet-feeding periods. After 2 weeks of the obesity-inducing period, HFD-fed mice showed significantly higher body weights than those of mice in the ND group, which were fed a normal diet. No significant differences in body weight were observed between HFD- and PSE-treated groups during the adaptive period. In contrast, during the following 2 weeks of the experimental diet-feeding period, the mice in the H-PSE (500 mg PSE/kg HFD) group retained significantly lower body weight than that of the mice in the HFD group (*p* < 0.05). Mice in the L-PSE (250 mg PSE/kg HFD) group had slightly lower body weights than those in the HFD group; however, the difference was not significant.

Figure 2b shows the cumulative weekly food intake of the mice in each group during the experimental period. At weeks 3 and 4, which corresponded to the two weeks of the adaptive period, mice in the HFD, L-PSE, and H-PSE groups exhibited a significantly lower daily food intake than that in ND group mice (*p* < 0.05). In the first week of the experimental diet period (week 5), both the L-PSE and H-PSE groups exhibited lower food intake than that of the ND group (*p* < 0.05), whereas no significant differences were observed among the groups in the last week (6 weeks). In both the adaptive and experimental diet periods, there was no significant difference in cumulative food intake between the HFD- and PSE-administered groups. During the experimental period, no symptoms of diarrhea or emesis were observed in any mouse.

### 2.2. Weight of Organs and Visceral Fat Tissues

Table 1 shows the body weights and weights of the liver, kidney, gastrointestinal tract, and visceral fat tissues of the mice in each group at the end of the experimental period. Mice in the HFD group had significantly higher body weights than those in the ND group, and body weight was significantly lower in the H-PSE group than that in the HFD group. Consistently, the perirenal, testicular, and total visceral fat masses of mice in the H-PSE group were significantly lower than those of mice in the HFD group. No significant differences in final body weight or visceral fat mass were observed between the HFD and L-PSE groups.

Kidney weights in the HFD, L-PSE, and H-PSE groups were significantly higher than those in the ND group (*p* < 0.05); however, there were no significant differences among the HFD, L-PSE, and H-PSE groups. Additionally, no significant differences were observed in the weights of the liver and gastrointestinal tract between the groups.

### 2.3. Histological Analysis of Small Intestine Tissue

**Figure 3:**
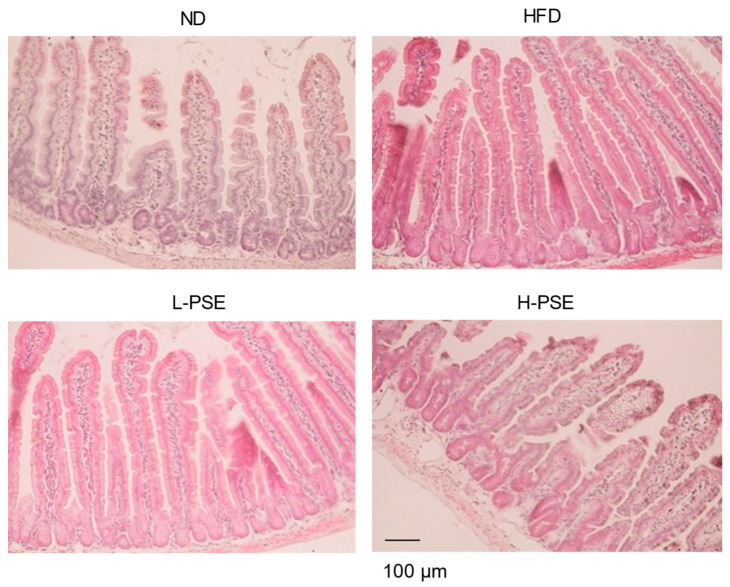
Histological structures of small intestine tissue. Hematoxylin and eosin (H&E) staining of paraffin-embedded upper jejunum tissue sections obtained from the mice in each group. Representative images from each group are shown. Scale bars correspond to 100 μm.

Subsequent examination of the internal organs of the mice following dissection revealed no instances of bleeding or adhesions within the gastrointestinal tract in any group. Figure 3 shows H&E staining of jejunal sections of mice in the experimental groups. Jejunal sections obtained from mice in each group exhibited normal villous structures. No evidence of small intestinal tissue damage, such as villous atrophy or significant inflammation, was observed. These results suggested that PSE administration did not cause inflammation or disruption of the jejunal architecture.

### 2.4. Blood Glucose, Plasma Lipids, and Total Liver Lipid Concentrations

The concentrations of fasting blood glucose, plasma lipids, and liver lipids in mice of each group are shown in Table 2. Mice in the HFD group had significantly higher blood glucose and plasma cholesterol concentrations than those in the ND group. PSE administration did not decrease blood glucose and plasma cholesterol levels compared with those in the HFD group. Regarding plasma triglyceride levels, no significant difference was observed between the ND and HFD groups; however, mice in the L-PSE group showed significantly lower triglyceride levels than those in the ND group. Additionally, there were no significant differences in the plasma NEFA levels among the groups. In contrast, the plasma norepinephrine concentrations in the HFD and L-PSE groups were one-fourth of those in the ND group (*p* = 0.050 and *p* = 0.040, respectively). The norepinephrine level was 2.9 times higher in the H-PSE group than that in the HFD group; however, the difference was not statistically significant due to a considerable variation in the H-PSE group (*p* = 0.347).

No significant differences in liver lipid concentrations were observed between the ND and HFD groups. However, mice in the H-PSE group exhibited a substantial reduction in liver lipid and cholesterol concentrations compared with those in the ND and HFD groups (*p* < 0.05).

### 2.5. Expression of Genes Involved in Triglyceride Metabolism in Adipose Tissue

**Figure 4:**
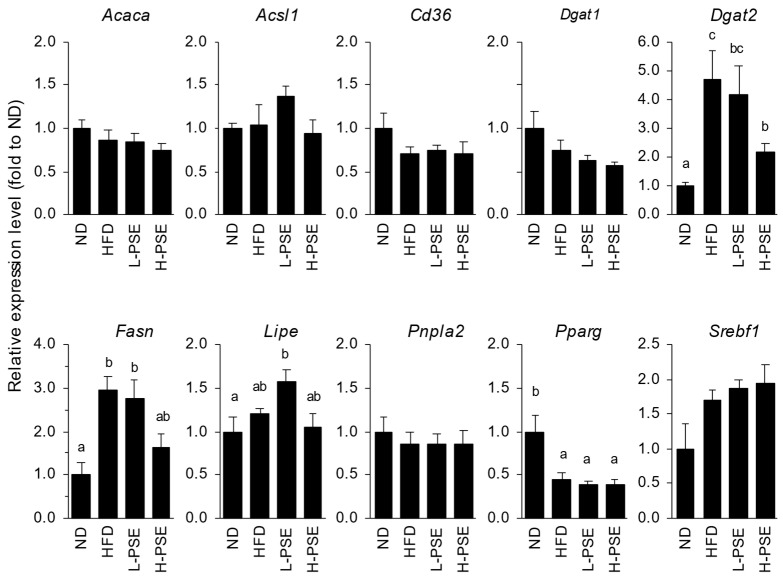
Expression levels of genes involved in lipid metabolism in the adipose tissue of mice in each group. The relative amounts of transcripts were normalized to those of *Actb* transcripts present in the same cDNA sample. Values are represented as folds of that obtained from the ND group. ^a,b,c^ Different letters indicate significant differences between values (*p* < 0.05, ANOVA followed by Tukey’s test). The absence of letters indicates that no significant differences were observed.

The expression levels of genes involved in triglyceride synthesis in testicular adipose tissue are shown in Figure 4. The HFD significantly increased the expression levels of *Fasn* (encoding fatty acid synthase, FAS) and *Dgat2* (encoding diglyceride acyltransferase 2, DGAT2) and decreased *Pparg* (encoding peroxisome PPAR-γ). The administration of high-dose PSE significantly decreased the expression of *Dgat2* (*p* = 0.012) and moderately decreased the expression of *Fasn* (*p* = 0.059). Mice in the L-PSE group showed significantly higher expression of *Lipe* (encoding hormone-sensitive lipase, HSL) than those in the ND group but not in the H-PSE group.

### 2.6. Expression of Adipocytokine Genes in Adipose Tissue

**Figure 5:**
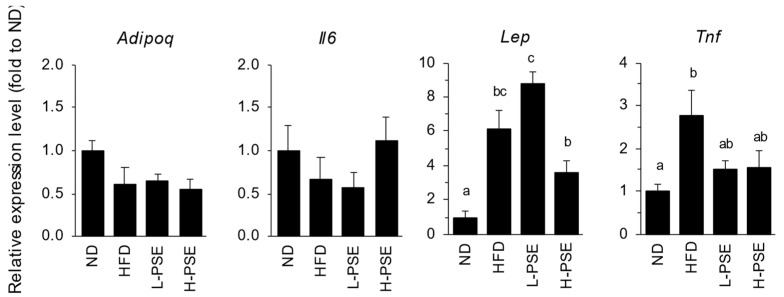
Expression levels of genes for adipocytokines in adipose tissue of mice in each group. The relative amounts of transcripts were normalized to those of *Actb* transcripts present in the same cDNA sample. Values are represented as folds of that obtained from the ND group. ^a,b,c^ Different letters indicate significant differences between values (*p* < 0.05, ANOVA followed by Tukey’s test). The absence of letters indicates that no significant differences were observed.

White adipose cells produce and secrete adipocytokines. Figure 5 shows the gene expression levels of adipocytokines in the adipose tissue of mice in each group. The expression levels of *Lep* (encoding leptin) and *Tnf* (encoding tumor necrosis factor α, TNF-α) genes in the HFD group were 6 and 2.8 times those in the ND group, respectively (*p* < 0.05). The expression of *Lep* and *Tnf* in the H-PSE group was lower than that in the HFD group, although the differences were not significant (*p* = 0.101 and 0.161, respectively). No significant differences were observed among the groups in the *Adipoq* (encoding adiponectin) and *Il6* (encoding interleukin-6) gene expression levels.

## 3. Discussion

In the present study, we revealed that PSE administration has a preventive effect on HFD-induced obesity in mice. The administration of high-dose PSE (500 mg PSE/kg HFD, H-PSE group) resulted in a decrease in body weight gain and visceral fat accumulation, particularly in the testicular fat tissue. In contrast, the low-dose PSE group (250 mg PSE/kg HFD, L-PSE group) showed limited weight loss during the experimental period. PSE administration did not result in substantial changes in food intake. These findings indicate that PSE has the potential to suppress body weight gain induced by HFD in a dose-dependent manner, and this action is independent of the amount of food intake.

Pawpaw seeds contain various acetogenins, phytochemical polyphenols, and alkaloids. Annonaceous acetogenins act as strong inhibitors of the NADH dehydrogenase activity of complex I, decrease ATP production in the mitochondria [29,30,31], and have cytotoxic and neurotoxic properties [14,29,32,33,34,35]. However, during the experimental period, no behavioral abnormalities or gastrointestinal symptoms, such as diarrhea or emesis, were observed. Additionally, no significant differences were observed in the gastrointestinal tract weight among the groups (Table 1). Histological analysis of small intestinal tissue sections revealed no structural abnormalities (Figure 3). These observations suggest that the reduction in body weight gain observed with PSE administration is unlikely to be due to impaired nutrient absorption resulting from intestinal inflammation. However, as the present study did not directly evaluate the effects of PSE on nutrient absorption in the gastrointestinal tract, further research is necessary to ascertain whether PSE influences intestinal nutrient absorption.

Fasting blood glucose and total cholesterol levels in mice in the HFD group were significantly higher than those in the ND group, indicating the development of insulin resistance and hypercholesterolemia as a result of obesity induced by HFD consumption. PSE contains a variety of phytochemicals, including alkaloids and polyphenols, which have antioxidant activities similar to those of graviola extract [2,3,4,5,26]. Phenolic compounds in graviola exert beneficial effects on blood glucose regulation and lipid metabolism [18,22,25]. However, in the present study, PSE administration did not exert direct hypoglycemic or cholesterol-lowering effects despite its anti-obesity effects. This difference may be attributed to the low concentration and short duration of PSE administration in this study. Sasso et al. evaluated the effects of three oral doses (50, 100, and 150 mg/kg body weight) of aqueous graviola leaf extract administered to obese C57BL/6 mice over 12 weeks [25]. The administration of graviola leaf extract resulted in a dose-dependent reduction in body weight, plasma levels of LDL cholesterol, VLDL cholesterol, triglycerides, and the atherogenic index in a dose-dependent manner, whereas no acute toxicity was observed at a higher concentration (2000 mg/kg body weight). In the present study, experimental diets containing 250 mg (L-PSE group) or 500 mg (H-PSE group) of PSE per kg of HFD were provided for only two weeks at the final concentration, subsequent to HFD-induced obesity. Mice in the H-PSE group were estimated to receive approximately 50 mg of PSE per kg of body weight per day, based on their food consumption and body weight. Additionally, the concentration of phenolic compounds in the PSE used in this study was relatively low, with an estimated value of 11.0 mg gallic acid equivalent/g PSE, as compared to the values reported in other studies [1,2,7,8]. Consequently, given the relatively low dose of PSE used in this study, further studies using prolonged treatment durations and varying administration protocols are necessary to elucidate the potential of PSE in enhancing glucose and lipid metabolism.

Notably, the H-PSE group also exhibited significantly reduced hepatic lipid accumulation, including liver triglycerides and cholesterol, compared with the ND and HFD groups (Table 2). However, under the conditions used in this study, the progression of fatty liver was not evident in the HFD group, and there were no significant differences in blood triglyceride levels between the ND and HFD groups. Conversely, the levels of plasma norepinephrine, a hormone implicated in energy metabolism and lipolysis, decreased following HFD consumption (Table 2). The plasma norepinephrine level in the H-PSE group exhibited a 2.9-fold increase compared with the levels in the HFD group. However, given the absence of significant differences in plasma NEFA levels among the experimental groups, the influence of plasma norepinephrine appears to be limited.

Ingestion of HFD resulted in substantial increases in perirenal, testicular, and total visceral fat mass in mice (Table 1). Consistently, the expression levels of *Fasn* and *Dgat2* in the testicular adipose tissue of HFD mice were found to be several-fold higher than those in the ND group mice (3.0 and 4.7-fold, respectively). In addition, the expression levels of these genes in the H-PSE group were approximately half those observed in the HFD group (Figure 4). FAS and DGAT2 are key enzymes in fatty acid synthesis and the final step of triglyceride synthesis, respectively. The expression profiles of the aforementioned genes correlated with the final body weight and visceral fat mass among the groups. In contrast, the expression of HSL, a pivotal enzyme in lipolysis, did not differ substantially between the HFD and H-PSE groups. These findings suggest that the administration of PSE reduced the synthesis of fatty acids and triglycerides, which contributed to a decrease in visceral adiposity and body weight in obese mice, but did not enhance lipolysis via HSL.

With respect to adipocytokines, the expression of *Lep* (encoding leptin), a pro-inflammatory appetite-suppressing adipokine, was significantly higher in the HFD and L-PSE groups than that in the ND group (*p* < 0.05; Figure 5). It is widely acknowledged that leptin secretion is associated with visceral fat mass [36,37,38]. The elevated expression of leptin in the HFD and L-PSE groups may be a consequence of increased visceral fat mass. In contrast, the expression of leptin in the H-PSE group of mice exhibited a decreased expression in comparison with the levels observed in the mice of the HFD (*p* = 0.101) and L-PSE (*p* < 0.001) groups, reflecting their decreased visceral fat mass (Figure 5 and Table 1). The absence of an increase in food consumption in the H-PSE group suggested that the elevated leptin expression observed in this group was a secondary response to decreased visceral fat mass. Furthermore, the expression of TNF-α (*Tnf* gene), a pro-inflammatory cytokine associated with insulin resistance, was significantly increased in the HFD group compared with that in the ND group. TNF-α is an inflammatory adipocytokine that is closely linked to obesity-induced insulin resistance and increases in response to accumulation of visceral fat [39,40,41]. Despite the absence of statistically significant differences, both the L-PSE and H-PSE groups exhibited a suppression of TNF-α expression that was approximately half of that observed in the HFD group. However, fasting blood glucose levels remained unchanged following PSE administration; therefore, prolonged administration of PSE may yield more pronounced metabolic effects.

We previously reported that PSE treatment inhibits adipocyte differentiation and triglyceride accumulation in differentiated 3T3-L1 cells under glucose-restricted conditions [27]. The addition of PSE significantly decreased the expression levels of regulators involved in adipocyte differentiation and adipogenesis, including PPAR-γ, C/EBP-α, and SREBP−1c, in low-glucose medium. PSE treatment also increased lactate production and decreased the NAD^+^/NADH ratio in undifferentiated 3T3-L1 cells due to the energy depletion as a consequence of reduced oxidative phosphorylation in the mitochondria. Lee et al. also reported that pawpaw fruit extract with 70% ethanol suppressed adipocyte differentiation and lipogenesis-related protein expression in 3T3-L1 cells [28]. In their study, treatment with ethanolic fruit extracts inhibited the differentiation of 3T3-L1 preadipocytes and lipid synthesis by reducing the levels of proteins related to adipocyte differentiation and adipogenesis, including FAS, SREBP1, PPAR-γ, C/EBP-α, and adiponectin. However, in the present study the expression levels of regulator genes for adipogenesis, including SREBP−1c and PPAR-γ, were unaffected by the PSE administration, despite the decreased expression of FAS and DGAT2 (Figure 3). These observations suggest that PSE may suppress FAS expression independent of SREBP−1c and PPAR-γ signaling. At present, the mechanisms and signaling pathways through which PSE reduces the expression of these genes involved in fat synthesis remain to be elucidated. Since polyphenols and acetogenins modulate diverse signaling pathways, the bioactive compounds in PSE may influence signal transduction or epigenetics in adipose tissue. Alternatively, the reduced fat synthesis may be a secondary response to decreased fat accumulation induced by PSE administration.

It should be noted that this study contains several limitations. First, as previously mentioned, the PSE administration period of the current study was brief, spanning a duration of only two weeks following a two-week adaptation period. Additionally, the relatively low dosage of PSE used in this study resulted in constraints on the efficacy of PSE administration. In a previous study, the administration of aqueous graviola leaf extract to obese mice over a period of 12 weeks resulted in a dose-dependent reduction in body weight and plasma lipid levels [25]. While the anti-obesity effects of PSE were evident in the present study, the efficacy of the PSE in addressing hypoglycemic and hypolipidemic effects should be ascertained at higher doses and over extended periods of administration time. Second, the present study has yet to fully elucidate the detailed mechanisms underlying the fat-reducing effects of PSE. In obese mice, the administration of high-dose PSE resulted in a decrease in the expression of a fatty acid synthase (FAS) and a triglyceride synthase (DGAT2) in the adipose tissue, concomitant with a reduction in fat mass. However, the unrelated expression levels of adipogenesis regulator genes suggest that PSE administration may not exert a direct inhibition of adipocyte differentiation or fat synthesis. Further research is necessary to elucidate the underlying mechanism of the fat-reducing effect of PSE, including its effects on nutrient absorption in the gastrointestinal tract. Finally, the composition of the administered PSE was not determined in this study. PSE contains a variety of phytochemicals, including alkaloids, polyphenols, and acetogenins. These phytochemicals have also been detected in the leaves, twigs, bark, and fruit of the pawpaw plant [1,2,7,8]. The identification and quantification of the major bioactive compounds present in the PSE will provide a foundation for comprehending its fat-reducing mechanisms.

## 4. Materials and Methods

### 4.1. Preparation of PSE

PSE was prepared as previously described [27]. The hulled pawpaw seeds were ground into a powder using a blender (TML180, Tescom Co. Ltd., Tokyo, Japan) and extracted with 10 volumes of ethanol by gentle rotation mixing for 24 h at room temperature using a tube rotator (TR-350, AS-ONE, Osaka, Japan). Then the mixture was centrifuged at 2610× *g* for 10 min at 25 °C and the supernatant was collected. The solvent was dried using evaporation in vacuo, and the residue was stored at −80 °C until use. The total phenolic content of PSE was determined by the Folin–Ciocalteu colorimetric method using gallic acid as a reference standard [42].

### 4.2. Animals and Experimental Diet

Male C57BL/6J JmsSlc mice (4 weeks old) were obtained from Japan SLC Inc. (Shizuoka, Japan). The mice were individually housed in an air-conditioned room maintained under a 12 h light/dark cycle. The experimental diets were prepared using a commercial HFD (HFD32, containing 30% safflower oil, CLEA, Tokyo, Japan). PSE was resuspended in a safflower oil/water/ethanol vehicle mixture (10:10:1) at the concentration of 1 g PSE/7.56 g vehicle mixture and added to the HFD at concentrations of 100 mg PSE/kg HFD (adaptive period), 250 mg PSE/kg HFD (L-PSE group), or 500 mg PSE/kg HFD (H-PSE group). The experimental diet administered to the HFD group contained an equal amount of vehicle mixture without PSE. Each experimental diet was adjusted with the vehicle mixture to yield 1.8 g of additional safflower oil per kg of HFD (equivalent to 0.5% total fat).

### 4.3. Experimental Design

Twenty-four mice were fed a commercial pelleted diet (CE-2-pellet) for a week. A schematic representation of the experimental design is illustrated in Figure 1. Six mice were assigned to the normal diet (ND) group and maintained on the CE-2 diet, whereas the remaining mice were fed the HFD for a period of two weeks to induce obesity (obesity-inducing period). The mice in the latter group were divided into three groups: HFD, L-PSE, and H-PSE groups (*n* = 6 each). Mice in the L-PSE and H-PSE groups were fed 100 mg PSE/kg HFD for one week, followed by 250 mg PSE/kg HFD during the second week (i.e., the adaptive period). The L-PSE and H-PSE groups were then fed 250 mg PSE/kg HFD (L-PSE group) or 500 mg PSE/kg HFD (H-PSE group) for two weeks (experimental diet period).

All mice were provided an experimental diet with free access, and the amount of the diet consumed by each mouse was monitored daily. The body weight of the mice was measured three times a week. At the end of the experimental period, blood samples were collected under anesthesia after fasting for 18 h. The plasma was immediately separated from the blood cells using centrifugation and stored at −20 °C until further analysis. The liver, kidney, testicular fat, mesenteric fat, and perirenal fat tissues were excised and weighed. All experiments were approved by the University of Niigata Prefecture Animal Ethics Committee (approval number 2402) and conducted in accordance with institutional guidelines.

### 4.4. Determination of Blood Glucose, Plasma Lipids, and Total Liver Lipid Concentrations

Total blood glucose levels were determined using a blood glucose meter (Nipro FreeStyle Freedom Lite; Nipro Co., Osaka, Japan). The levels of plasma triglycerides, total cholesterol, and non-esterified fatty acids (NEFA) were determined using the following enzymatic and colorimetric test kits: LabAssay Triglyceride, LabAssay Cholesterol and NEFA C-test Wako (FUJIFILM Wako Pure Chemical Co., Osaka, Japan).

Total liver lipids were extracted using the Folch method, followed by solvent evaporation and weighing of the residue. The lipids were dissolved in 1 mL of isopropanol, and the concentrations of triglycerides and total cholesterol were determined using the LabAssay Triglyceride kit and LabAssay Cholesterol kit, respectively.

Plasma norepinephrine concentrations were determined using an enzyme immunoassay kit according to the manufacturer’s instructions (Norepinephrine ELISA kit; Abnova Co., Taipei, Taiwan).

### 4.5. Preparation of cDNA Samples

Total RNA was extracted from tissue samples using Sepasol-RNA I Super G (Nacalai Tesque, Kyoto, Japan). Frozen tissue samples were homogenized in 1 mL of Sepasol, and total RNA was prepared according to the manufacturer’s protocol. cDNA was synthesized from random primers using a PrimeScript First Strand cDNA Synthesis Kit (Takara Bio Inc., Shiga, Japan).

### 4.6. Quantitative Analysis of Gene Expression Using Real-Time PCR

The primers used in this study are listed in Table 3. The relative abundance of transcripts in the cDNA samples was determined using real-time PCR with SYBR Premix Ex Taq II (Perfect Real Time; Takara Bio Inc.) and a PicoReal 96 real-time PCR system (Thermo Fisher Scientific, MA, USA). The reaction was performed using 40 cycles of denaturation at 95 °C for 5 s and extension at 60 °C for 30 s, and the results were analyzed using the delta delta Ct method. The specificity of the amplification was confirmed using melting curve analyses. Relative amounts of transcripts were normalized to those of *Actb* (encoding β-actin) transcripts in the same cDNA sample. The mean Ct values of the *Actb* in the cDNA samples derived from an equivalent amount of RNA were as follows: ND group, 16.6 ± 0.45; HFD group, 15.5 ± 0.62; L-PSE group, 15.4 ± 0.59; and H-PSE group, 16.0 ± 0.54.

### 4.7. Histological Analysis of Small Intestinal Tissue

A portion of the upper jejunum was excised and fixed in 4% paraformaldehyde for 24 h and embedded in paraffin for histological analysis. The paraffin-embedded tissues were sectioned into 5 µm thick slices and stained with hematoxylin and eosin (H&E) following standard deparaffinization and rehydration procedures. The slides were analyzed, and respective sections were captured at 200× magnification using a light microscope with a digital camera (CK30, Olympus Co., Tokyo, Japan).

### 4.8. Statistical Analysis

Statistical analyses were performed using the R software (v.4.5.0, R Project for Statistical Computing, Vienna, Austria). Normality of the data was analyzed using the Shapiro–Wilk test. For multiple comparisons of mean values, statistical significance was evaluated using analysis of variance (ANOVA) and Tukey’s honest significant difference test. Statistical significance was set at *p* < 0.05.

## 5. Conclusions

This is the first report indicating that PSE administration suppresses HFD-induced obesity in mice. PSE exhibited a dose-dependent effect on the reduction in body weight gain and body fat mass, independent of alterations in food intake. However, the present study did not fully elucidate the precise fat-reducing mechanisms of PSE or its efficacy on glucose and lipid metabolism. It is also crucial to ascertain the active ingredient in PSE responsible for the reduction in fat accumulation in mice. Despite these limitations, it is anticipated that future studies will elucidate the potential of PSE to counteract obesity in humans.

## Figures and Tables

**Table 1:** Body weights and organ weights of the mice in each group at the end of the experimental period.

	ND	HFD	L-PSE	H-PSE
Body weight (g)	25.3 ± 0.28 ^a^	34.0 ± 1.31 ^c^	32.4 ± 0.81 ^bc^	29.2 ± 0.59 ^b^
Liver (g)	0.92 ± 0.03	1.02 ± 0.08	0.90 ± 0.06	1.06 ± 0.15
Kidney (g)	0.29 ± 0.01 ^a^	0.34 ± 0.01 ^b^	0.34 ± 0.01 ^b^	0.33 ± 0.01 ^b^
Gastrointestinal tract (g)	1.31 ± 0.04	1.40 ± 0.07	1.39 ± 0.07	1.25 ± 0.06
Mesenteric fat (g)	0.18 ± 0.01 ^a^	0.58 ± 0.07 ^bc^	0.67 ± 0.06 ^c^	0.43 ± 0.03 ^b^
Perirenal fat (g)	0.05 ± 0.01 ^a^	0.57 ± 0.07 ^c^	0.46 ± 0.04 ^c^	0.26 ± 0.05 ^b^
Testicular fat (g)	0.25 ± 0.01 ^a^	1.45 ± 0.13 ^c^	1.18 ± 0.11 ^bc^	0.78 ± 0.13 ^b^
Total visceral fat (g)	0.47 ± 0.02 ^a^	2.60 ± 0.23 ^c^	2.30 ± 0.19 ^c^	1.46 ± 0.20 ^b^

Values are shown as the mean ± SEM obtained from 6 mice. ^a,b,c^ Different letters indicate significant differences between values (*p* < 0.05, ANOVA followed by Tukey’s test). The absence of letters indicates that no significant differences were observed.

**Table 2:** Fasting blood glucose, plasma lipid, and total liver lipid levels of the mice in each group.

	ND	HFD	L-PSE	H-PSE
Blood glucose (mg/100 mL)	103.8 ± 8.9 ^a^	181.0 ± 15.8 ^b^	178.7 ± 20.6 ^b^	190.2 ± 13.5 ^b^
Plasma cholesterol (mg/100 mL)	55.3 ± 2.5 ^a^	127.8 ± 10.1 ^b^	113.6 ± 14.7 ^b^	125.7 ± 6.4 ^b^
Plasma triglyceride (mg/100 mL)	81.8 ± 7.5 ^b^	63.9 ± 3.2 ^ab^	49.2 ± 2.1 ^a^	62.4 ± 4.4 ^ab^
Plasma non-esterified fatty acid (mEq/L)	1.19 ± 0.13	1.15 ± 0.16	0.99 ± 0.10	1.05 ± 0.06
Plasma norepinephrine (ng/mL)	3.35 ± 0.61 ^b^	0.82 ± 0.11 ^ab^	0.72 ± 0.12 ^a^	2.37 ± 1.12 ^ab^
Liver triglyceride (mg/g wet tissue)	68.7 ± 5.2 ^b^	60.1 ± 10.8 ^ab^	48.4 ± 5.4 ^ab^	35.2 ± 5.7 ^a^
Liver cholesterol (mg/g wet tissue)	5.2 ± 0.4 ^b^	5.4 ± 0.4 ^b^	4.7 ± 0.4 ^ab^	3.5 ± 0.3 ^a^
Total liver lipid (mg/g wet tissue)	147.0 ± 7.3 ^b^	139.7 ± 14.6 ^b^	117.0 ± 6.8 ^ab^	92.4 ± 6.0 ^a^

Values are shown as the mean ± SEM obtained from 6 mice. ^a,b^ Different letters indicate significant differences between values (*p* < 0.05, ANOVA followed by Tukey’s test). The absence of letters indicates that no significant differences were observed.

**Table 3:** Primers used in this study.

Gene	Forward Primer (5’–3’)	Reverse Primer (5’–3’)	Accession Number
*Actb*	cttgggtatggaatcctgtgg	gtacttgcgctcaggaggag	NM_007393
*Acaca*	gcaactgacagaggaagatgg	tggaaggggaatccatagtg	NM_133360
*Adipoq*	cccagtcatgccgaagatga	agtgccatctctgccatcac	NM_009605
*Acsl1*	agatggctggttacacacgg	taggctctcaacgtcgggta	NM_001302163
*Cd36*	gtgcaaaacccagatgacgt	tccaacagacagtgaaggct	NM_001159558
*Dgat1*	atatccccgtgcacaagtgg	agaatcggcccacaatccag	NM_010046
*Dgat2*	ggcgctacttccgagactac	tccggaagttaccagccaac	NM_026384
*Fasn*	tctgtgcccgtcgtctatac	ggaggtatgctcgcttctct	NM_007988
*Il6*	cggccttccctacttcacaa	caagtgcatcatcgttgttca	NM_031168
*Lep*	gacatttcacacacgcagtcg	agcccaggaatgaagtccaa	NM_008493
*Lipe*	tgagattgaggtgctgtcgt	gtaccttgctgtcctgtcct	NM_010719
*Pnpla2*	caacgccactcacatctacg	accaggttgaaggagggatg	NM_001163689
*Pparg*	agggcgatcttgacaggaaa	cgaaactggcacccttgaaa	NM_001127330
*Srebf1*	cccacctcaaacctggatct	aagcagcaagatgtcctcct	NM_011480
*Tnf*	cacagaaagcatgatccgcg	actgatgagagggaggccat	NM_001278601

Genes encoding for: *Actb*, β-actin; *Acaca*, acetyl-CoA carboxylase 1 (ACC1); *Acsl1*, acyl-CoA synthetase 1 (ACS1); *Adipoq*, adiponectin; *Cd36*, CD36 molecule; *Dgat1*, diglyceride acyltransferase 1 (DGAT1); *Dgat2*, diglyceride acyltransferase 2 (DGAT2); *Fasn*, fatty acid synthase (FAS); *Il6*, interleukin 6; *Lep*, leptin; *Lipe*, hormone sensitive lipase (HSL); *Pnpla2*, adipose triglyceride lipase (ATGL); *Pparg*, peroxisome proliferator-activated receptor γ (PPAR-γ); *Srebf1*, sterol regulatory element binding protein 1c (SREBP-1c); *Tnf*, tumor necrosis factor α (TNF-α).

## Data Availability

The original contributions presented in this study are included in the article. Further inquiries can be directed to the corresponding author.

## Abbreviations

The following abbreviations are used in this manuscript:

ANOVA	analysis of variance
C/EBP	CCTTA/enhancer-binding protein
DGAT2	diglyceride acyltransferase 2
FAS	fatty acid synthase
H&E	hematoxylin and eosin
HFD	high-fat diet
HSL	hormone-sensitive lipase
NEFA	non-esterified fatty acids
ND	normal diet
PSE	pawpaw seed extract
PPAR	proliferator-activated receptor
SREBP	sterol regulatory element-binding protein
TNF	tumor necrosis factor
